# Trends in induced abortion among Nordic women aged 40-44 years

**DOI:** 10.1186/1742-4755-8-23

**Published:** 2011-08-16

**Authors:** Adam Sydsjö, Ann Josefsson, Marie Bladh, Gunilla Sydsjö

**Affiliations:** 1 Division of Obstetrics and Gynecology, Department of Clinical and Experimental Medicine, Faculty of Health Sciences, Linköping University, SE-581 83 Linköping, Sweden; 2 Department of Obstetrics and Gynecology in Linköping, County Council of Östergötland, Se- 581 85 Linköping, Sweden

**Keywords:** induced abortion, age, contraceptive, abortion rates

## Abstract

**Objectives:**

Women aged 40-44 years in 2005 ought to have been subjected to much more influence on attitudes and knowledge on contraceptive methods during their fertile period than women who were in the same age span in 1975 when the abortion laws were introduced.

**Material:**

From official statistics, the rates of induced abortion and birth rates in women aged 40-44 years were collected for Sweden, Denmark, Norway and Finland for each five-year during the period 1975-2005.

**Results:**

With the exception of Sweden all other studied Scandinavian countries have lowered their abortion rates since 1975 (p < 0.001) and reduced the proportion of induced abortions in relation to birth rate (p < 0.001). In 2005 these countries also had lower rates of induced abortion than Sweden in the age group 40-44 years (p < 0.001).

**Conclusion:**

There is a significant change in rates of induced abortion in women aged 40-44 years in Finland, Norway, Denmark, and at status quo in Sweden. 40-44 years in Finland, Norway, Denmark, and at status quo in Sweden. This indicates that family planning programs works well in the Nordic countries. The differences found may be assumed to possible diverging focus on attitudes or ethical considerations.

## Introduction

Induced abortion is a procedure made lawful by several societies, especially in the western world [1]. Still it is prohibited for cultural or religious reasons in many parts of the world, frequently leading to terrible consequences for women involved, as they are forced to consult facilities not lead by professionals, often leading to severe complications or even maternal death [2-4].

A main reason to legalise induced abortion was to provide a safe procedure for the pregnant woman with an unplanned pregnancy and not force her to seek help from unprofessional persons or to have to travel abroad to countries, in which induced abortion was legal at that time, but medically unsafe [5].

In the Nordic countries, induced abortion has been legal since the mid seventies [6], but the societies also stressed the need for adequate family planning facilities in order to avoid unplanned pregnancies [6]. All Nordic countries are regarded as modern well-fare states and are often used as role models in many social aspects. Support for families in these countries, for instance in connection with pregnancy and childbirth, is well recognised and quite substantial [7,8].

Most studies on induced abortion have ended in an assumption that increased support automatically leads to visible results on abortion rates [9,10]. Lack of such support is an explanatory factor for high rates of abortion in developing countries [11]. Such economic explanations were used to elucidate the high rates of induced abortion in former eastern bloc countries. However, in the nineties as economies improved, most of these countries have established lower abortion rates [12]. For that reason one should expect rates of induced abortions to fall, as more support was added over time in family planning programs.

To study what must be considered as reasonable proportions or changes in rates of induced abortions, straight comparisons between societies with similar family planning programs may be of help. The Scandinavian countries seem ideal, as they have so much in common e.g. health care, religion and culture [7,8].

A question arising in this context is, how a society can measure or estimate if it has achieved a success in its efforts, as it is impossible to estimate this on an individual level and an individuals' behaviour may shift over time, depending on family situation, age and fertility. Ideally, women at the end of their fertile period, namely 40-44 years should be well suited to investigate in this aspect, as this is a group of women who have been subjected to health care and family planning program during most part of their fertile life. By establishing attitudes through life and using available facilities, rates of induced abortion hypothetical ought to diminish in this age group over time.

Nordic women in the age span 40 - 44 years have presumably been subjected to a much more extended education in family planning and easy access to a variety of contraceptive methods during their fertile period than women who were in the same age span in the 1970-1980s when the abortion laws were introduced. We therefore decided to study the abortion rates in this age group. We also investigated the proportions of induced abortion in relation to birth rates as this might be used as a blunt indicator on how well a nation's family planning system is actually applied.

## Method

The data in this study is derived from official statistics as presented in a joint venture by the Nordic governments (Denmark, Norway, Sweden and Finland) and health care providers [13,14]. We have chosen to omit Iceland, as the population of Iceland is merely about 300 000 inhabitants.

The female age group 40-44 was followed for each five year period from 1975 to 2005 in order to determine the rate of induced abortions and birth rate per 1000 women in the same age group. The rates were then finally used to calculate the proportion of all conceived pregnancies that ended in induced abortion.

As laws regulating abortion were not introduced in Norway until 1978 [15], we used 1980 as the start point for Norway, since the figures for the rest of the period still provide vital information on trends.

Figures on population were collected from each countries official database on population in order to statistically validate differences in abortion rates as well as in number of abortions between the Nordic countries by means of Chi-2 tests. All statistical analyses were performed using SPSS, version 16.0 (SPSS Inc., Chicago, IL.).

## Results

In all countries the birth rate was higher in 2005 than in 1975.

**Figure 1:**
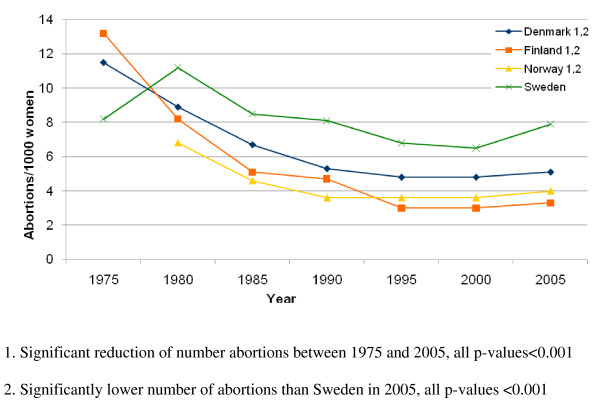
**Number of induced abortions on women aged 40-44 years**.

Figure 1 show how the rates of induced abortion have developed over time among women aged 40-44 years. All countries with the exception of Sweden have significantly lowered their abortion rates since 1975 (p < 0.001). In 2005 all countries had lower rates of induced abortion than Sweden in the age group 40-44 years (p < 0.001).

**Figure 2:**
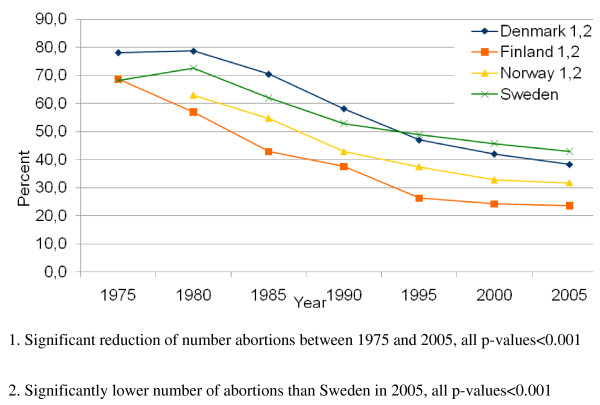
**Number of abortions in relations to number of pregnancies for women aged 40-44 years**.

The same development is also found for the proportion of induced abortions in relation to childbirth, Figure 2. With the exception of Sweden, the Scandinavian countries had reduced the proportion of induced abortions in relation to birth rate (p < 0.001) and in 2005 they all had significantly lower proportions as compared to Sweden (p < 0.001).

## Discussion

In this study we found that the rate of induced abortion receded significantly in all countries but Sweden, where the decrease was marginal. Also when the rates of induced abortion and birth rates were compared, the same trend was found. Thus in Finland only 25% induced abortions in relation to born infant took place, a significant reduction as compared to the proportion in 1975 [13]. The reduction in Finland from 70% to 25% suggests that pregnancies in this age span are more wanted and planned and that Family planning is well functioning.

As rates of induced abortion among women aged 40-44 years are relatively small compared to younger women, this group of women has to our knowledge so far not been focused on in the literature. Our study shows that even if the rate of abortion is small, compared to the younger age groups, quite substantial changes have taken place. We interpret this observation as a result of the influences of society to form attitudes during a woman's reproductive age to motivate her to plan her childbearing. It is most likely that a woman in this age group in 2005 should have been subjected to an easy available family planning system with a variety of modern contraceptives during her fertile period and as a result the rates of induced abortion are lower in 2005 than in 1975.

Theoretically, improved methods and introduction of screening programs for the detection of foetal malformations or chromosomal abnormalities over time should contribute to an increased rate of abortions in women aged 40-44 years [16]. The number of induced abortions due to malformations or chromosomal abnormalities, is however quite small in Sweden, only 497 of 37 000 induced abortions in 2006 [17], besides, the eventual impact of improved detection of foetal malformations is probably of no importance, as rates of induced abortion are actually receding.

A factor forming attitude in Sweden may well be, that abortion as a phenomenon is primarily stressed as a women's choice issue and is not very much debated. The lack of debate on induced abortion in Sweden has been explained as due to that the society has not wanted to put a personal blame on a woman's personal choice [18]. Induced abortion, however, is much debated if it comes to e.g. foetal malformations or Morbus Down, as it is considered unethical by some patient organisations [19,20]. Likewise, induced abortion because of female gender is raising opinions. In such cases the unborn foetus is regarded as a future human being in spite of its gender or handicap.

Maybe, the abortion procedure itself is so facilitated by the 18 weeks limit in Sweden, easy accessible and considered safe that a woman for that reason does not reflect to use contraceptives, even if such are easily accessible [10]. In contrast, in Finland a woman has to make a request for abortion. In both Norway and Denmark a request is necessary after 12 weeks of pregnancy, while in Sweden induced abortion is the woman's own decision up to the 18 weeks of pregnancy [6]. Such policies may explain the differences found in this study. As most Scandinavian women must apply for an abortion, at least after the 12^th ^week of pregnancy, this may make the women more aware to avoid an unwanted pregnancy. As there is no national register on induced abortions in Sweden such hypothesis is difficult to investigate, but re-abortion does not seem uncommon [18]. Still, such registers exist in the other Nordic countries.

As all included Scandinavian countries have ample funds allocated for health and welfare, it is unlikely that any of these countries lack in resources or cannot provide enough funds to cover an adequate family planning policy. It is also well known that the Swedish social benefits during pregnancy and after are childbirth is generous in an international perspective [5].

Although this is a small study based on information readily available in official statistics, it provides valuable information. Comparing rates of induced abortion between countries may in many aspects be difficult, as there are differences in traditions or religious influences in society. The advantage of comparing the Nordic countries is found in the homogeny structure of the countries with no main religious or political differences. An interesting aspect in this context may well be that authorities and politicians in Sweden any way may have different attitudes and perhaps more liberal attitudes to the issue than the rest of the population. Such questions are so far poorly investigated, but we have initiated such a study.

Fertility diminishes for natural reasons in women, but still 50% of the women may conceive well in to their 50s [21]. As societies do not cover the costs for women in this special age span, the influence of IVF treatment is probably negligent.

It is well known and reported that younger women are the group, which most frequently use oral contraceptives. As the major part of induced abortions is performed on younger women, the efforts from society to provide older women with safe contraceptive methods may therefore be less. An explanation may be, that are differences in the use of intrauterine devises (IUD) between the four Scandinavian countries [7,8,15]. As IUDs might be seen as a preferable method among women who have given birth, a country using more IUDs is better off in the aspect of contraception. Also, in some parts of Sweden, the counties have started to take a quite substantial fee for performing legal sterilisation, which may contribute to the higher rates of induced abortion among women 40-44 years old.

It has been observed, that women who recently have immigrated to a country have a more frequent rate of induced abortions that women born in the country itself [22-24]. As Sweden has a higher proportion of refugees and immigrants, this may in some aspect explain the difference to the other Nordic countries. For instance, Finland with a very low inflow of refugees also has the lowest rates of induced abortions. It must be remembered, that for this cultural reason it may be difficult to apply supportive and traditional family planning as used on a more traditional Swedish population.

## Conclusion

The findings in this study show that there is a significant change in rates of induced abortion in women aged 40-44 years in Finland, Norway, Denmark, and at status quo in Sweden. This indicates that family planning programs works well in the Nordic countries. The differences found may be assumed to possible diverging focus on attitudes or ethical considerations.

## Competing interests

The authors declare that they have no competing interests.

## Authors' contributions

AS; Research idea, design, preparation of the manuscript, responsible for the final preparation of the manuscript AJ; Design, preparation of the manuscript, analysis of data MB; Data collection, design, preparation of the manuscript, analysis of data. GS; Data collection, design, preparation of the manuscript, responsible for the final preparation of the manuscript. All authors have read and approved the final version of the manuscript.

## References

[B1] Cook RJ Dickens BM Bliss LE International developments in abortion law from 1988 to 1998 Am J Public Health 1999 89 579 86 10.2105/AJPH.89.4.579 10191808 PMC1508897

[B2] Mayor S Pregnancy and childbirth are leading causes of death in teenage girls in developing countries BMJ 2004 328 1152 15142897 10.1136/bmj.328.7449.1152-a PMC411126

[B3] Singh S Hospital admissions resulting from unsafe abortion: estimates from 13 developing countries Lancet 2006 368 9550 1887 1892 10.1016/S0140-6736(06)69778-X 17126721

[B4] World Health Organization Unsafe abortion: Global and regional estimates of the incidence of unsafe abortion and associated mortality in 2003 2007 5 Geneva: World Health Organization

[B5] Sundström K Family planning in Sweden during 100 years. [Abortion and contraceptive agents- from law-breaking to reproductive right] Lakartidningen 2004 101 88 93 14763010

[B6] Knudsen LB Gissler M Bender SS Hedberg C Ollendorf U Sundström K Totlandsdal K Vilhjalmdottir Induced abortion in the Nordic countries: special emphasis on young women Acta Obstet Gynecol Scand 2003 82 257 268 10.1034/j.1600-0412.2003.00006.x 12694123

[B7] Goldstein H Legal abortion in Denmark during the past 25 years: aspects of public health and ethics Eur J Contracept Reprod Health Care 1998 3 3 155 9 9853207 10.3109/13625189809051419

[B8] Mandelin MA Pregnancy termination-situation in Finland Acta Obstet Gynecol Scand Suppl 1997 164 51 3 9225638

[B9] Rahm VA Subsidized pills to teenagers: a 1-year trial in Gävle Lakartidningen 1991 19 88 2296 2297 2062134

[B10] Persson E Gustafsson B Van Rooijen M Reduced number of abortions: Result of easily accessible and qualitative contraception counselling and cheap oral contraceptives Lakartidningen 1994 91 4097 4100 7808107

[B11] United Nations; Department of Economic and Social Affaires; Population Division World Abortion Policies 2007 2007 New York: United Nations

[B12] Sedgh G Henshaw S Singh S Ahman E Shah IH Induced abortion: estimated rates and trends worldwide Lancet 2007 370 1338 1345 10.1016/S0140-6736(07)61575-X 17933648

[B13] Induced abortions in the Nordic countries 2009 2011 http://www.stakes.fi/tilastot/tilastotiedotteet/2011/Tr09_11.pdf

[B14] Health Statistics in the Nordic Countries with data from 2008 http://nomesco-eng.nom-nos.dk/filer/publikationer/Helsestatistik2010.pdf

[B15] Lökeland M Abortion: the legal right has been won, but not the moral right Reprod Health Matters 2004 12 24 Suppl 167 73 15938170 10.1016/s0968-8080(04)24016-2

[B16] Fetal diagnostics and abortion. Proposition 1994/95:142 Ministry of Health and Social Affairs Stockholm 1995

[B17] The National Board of Health and Welfare Official Statistics of Sweden Health and Diseases. 2006 2007 Stockholm

[B18] Kero A Hogberg U Jacobsson L Lalos A Legal abortion: a painful necessity Soc Sci Med 2001 53 1481 1490 10.1016/S0277-9536(00)00436-6 11710423

[B19] de la Fuente Fonnest I Sondergaard F Fonnest G Vedsted-Jacobsen A Attitudes among health care professionals on the ethics of assisted reproductive technologies and legal abortion Acta Obstet Gynecol Scand 2000 79 49 53 10.1080/j.1600-0412.2000.079001049.x 10646816

[B20] Hammarstedt M Jacobsson L Wulff M Lalos A Views of midwives and gynecologists on legal abortion: a population-based study Acta Obstet Gynecol Scand 2005 84 58 64 15603569 10.1111/j.0001-6349.2005.00695.x

[B21] Blaney CL Contraceptive needs after age 40 Network 1997 18 4 7 12321066

[B22] Helstrom L Zatterstrom C Odlind V Abortion rate and contraceptive practices in immigrant and Swedish adolescents J Pediatr Adolesc Gynecol 2006 19 209 213 10.1016/j.jpag.2006.02.007 16731415

[B23] Helstrom L Odlind V Zatterstrom C Johansson M Granath F Correia N Abortion rate and contraceptive practices in immigrant and native women in Sweden Scand J Public Health 2003 31 405 410 10.1080/14034940210165181 14675931

[B24] Vangen S Eskild A Forsen L Termination of pregnancy according to immigration status: a population-based registry linkage study BJOG 2008 115 1309 15 10.1111/j.1471-0528.2008.01832.x 18715418

